# Editorial: Microenvironment-derived stem cell plasticity—volume II

**DOI:** 10.3389/fcell.2022.967461

**Published:** 2022-09-14

**Authors:** Ivana Gadjanski, Slavko Mojsilovic, Marietta Herrmann, Jelena Krstic

**Affiliations:** ^1^ Biosense Institute, Center for Biosystems, University of Novi Sad, Novi Sad, Serbia; ^2^ Institute for Medical Research, University of Belgrade, Belgrade, Serbia; ^3^ IZKF Group Tissue Regeneration in Musculoskeletal Diseases, University Hospital Würzburg, Würzburg, Germany; ^4^ Bernhard-Heine Center for Locomotion Research, University of Würzburg, Würzburg, Germany; ^5^ Division of Cell Biology, Histology and Embryology, Gottfried Schatz Research Center, Medical University of Graz, Graz, Austria

**Keywords:** plasticity, microenvironment, stem cell therapy, 3D culture, cancer stem cells, neural stem cells, immunity

Microenvironment or a niche is a very complex and dynamic system that has multiple biochemical and mechanical effects on the residing cells, guiding their maturation, differentiation, cell-cell interactions and homeostasis, as well as their response to pathophysiological conditions. When considering stem cells (SCs), such a SC niche is a major provider of various cues that influence the cells’ phenotypic potential. In the second volume of this Research Topic contributors presented different examples of how specific microenvironmental effects can be harnessed in developing therapeutic approaches for metastatic bone cancer and diseases of the central nervous system (CNS), as well as emphasizing immune-centric and niche-based targeting. One contribution provides information on importance of a biomimetic *in vitro* microenviroment for designing models of both physiological and pathophysiological conditions.

In their original research study Di Pompo et al. present results on development of a potential therapy against metastatic bone osteolysis. In order to metastasize, cancer cells (CC) have to survive their detachment from the primary tumor tissue. This mostly affects dampening of the increased rates of reactive oxygen species and ability to increase the intracellular NADPH levels (Elia et al., 2018). Once the detached CCs avoid anoikis, they need to adapt to a new microenvironment and acquire mechanisms to support their proliferation (Elia et al., 2018). Metastasis to the bone requires extracellular matrix (ECM) remodeling and depends on the activity of differentiated osteoclasts. Di Pompo et al. demonstrate that increased proton excretion produced by glycolytic breast CCs, increases acidity of the microenvironment, thus promoting secretion of pro-osteoclastogenic factors (e.g., RANKL and IL-8) by osteoblasts and inducing resident osteoclast precursors to migrate and differentiate into osteoclasts. This further degrades bone tissue and allows further penetrance of CCs. The study is mostly based on *in vitro* data, while data from xenograft models and patient bone metastasis, showing significant correlation of the osteolytic marker TRACP5b with IL-8, is also provided. Di Pompo et al. suggest neutralizing antibodies against IL-6 or IL-8 as a potential therapy against metastatic bone osteolysis. However, it would also be fascinating to investigate whether bone metastasis could be inhibited by modifying the local microenvironment through cancer metabolism targeting (especially glycolysis).

Following the topic of harnessing the environmental effects for therapeutic purposes, Berlet et al. present a literature review in which they discuss different regenerative therapies as possible treatment options for CNS disorders, such as stroke, Parkinson’s and Huntington’s diseases. These might involve SC therapies, utilizing embryonic SCs (ESCs), induced pluripotent SCs (IPSCs), neuronal progenitor SCs (NSCs) and/or mesenchymal SCs (MSCs) building on their potential to reconstruct the neural circuitry or bystander effects to modulate the immune response. Berlet et al. provide a comprehensive overview on regenerative medicine for treating CNS disorders, focusing on discussing how these strategies can be improved by an enriched environment and exercise. The literature review emphasizes that such an approach can be indeed of great therapeutic value, by improving survival and functional integration of immature cell grafts within the host environment as well as stimulating endogenous repair mechanisms. This was found to be predominantly relevant for stroke and Huntington’s disease. Overall, this article emphasizes the importance of the host environment for therapeutic success of SC therapies.

However, a discussion on environmental factors in tissues cannot be developed without talking about inflammation. One could argue that there is practically no tissue dynamics without some form of inflammatory process (Medzhitov, 2008). In a wider sense, inflammation and some level of involvement of the immune system is in the essence of both physiologic and pathologic processes, including homeostasis maintenance, tissue regeneration, aging, degenerative, metabolic, and malignant diseases, apart fighting infections. In their review paper, Garg et al. analyze the role of inflammation in tissue regeneration, aiming to provide an integrated view, based on analysis of ample relevant literature, on how crosstalk between components of the immune system and SCs in tissue niches impacts tissue behavior during development, aging, and injury. In a complex and comprehensive essay, the authors lead us through different aspects of innate immunity (alarmins, immune cells, complement system) and their impact on SC fate decision. They further discuss the role of inflammaging, a chronic low grade systemic inflammation associated with aging, and a phenomenon of inflammation-triggered transdifferentiation in homeostasis and stress response. Finally, the authors conclude with the call for further research to expand our knowledge and understanding of intricate tissue niche-specific factors influencing SC decisions in order to envisage immune-centric and niche-based targeting approaches for novel regenerative therapeutic strategies.

A call for further research towards more precise and accurate 3D model systems that can mimic *in vivo* microenvironment, both in physiological and pathophysiological conditions, is also voiced by Flagelli et al. in their interesting research report. As emphasized earlier, *in vivo* microenvironment is a very complex system, particularly so for CNS that is composed of various cell types. 2D models have a limited ability to provide adequate biomimetic analog of a 3D CNS microenvironment which is why extensive research efforts are being directed towards development of 3D models that could recapitulate more efficiently various stimuli found *in vivo* (Yildirimer et al., 2019). In their study Flagelli et al. present a novel 3D culture device designed specifically for stimulation of oligodendrocyte precursor cells (OPCs) differentiation from multipotent NSCs, towards premyelinating and mature myelinating oligodendrocytes (OLs). The device provides a new 3D culture microenvironment, based on the fiber-matrix made of PBT, an inert and biocompatible synthetic polyester. Such fiber mesh was functionalized with a laminin coating, as laminin is one of the main ECM components thus further increasing the mimicking of the NSCs’ *in vivo* niche. In such a device, mechanical and biochemical cues provided by the fibers and laminin-coating, strongly accelerated maturation of OPCs and their differentiation to OLs that even spontaneously wrapped the fibers in a myelination-like morphology. It will be exciting to see further developments of this device prototype towards a microfluidic system and eventually a bioreactor that could be used as a preclinical platform for axon myelination assays and drug testing in demyelinating diseases.

Overall, this Research Topic once more confirms the importance of the microenvironment for disease development, sustainability and treatment and points to the yet unexplored areas of research in the field.
